# Cognitive impairment in multiple sclerosis: “classic” knowledge and recent acquisitions

**DOI:** 10.1055/s-0043-1763485

**Published:** 2023-06-28

**Authors:** Chiara Piacentini, Ornella Argento, Ugo Nocentini

**Affiliations:** 1 Institute of Hospitalization and Care of a Scientific Character “Santa Lucia” Foundation, Behavioral Neuropsychology, Rome, Italy.; 2 University of Rome “Tor Vergata”, Department of Clinical Sciences and Translational Medicine, Rome, Italy.

**Keywords:** Multiple Sclerosis, Cognitive Dysfunction, Social Cognition, Decision Making, Neuropsychological Tests, Morale, Esclerose Múltipla, Disfunção Cognitiva, Cognição Social, Tomada de Decisões, Testes Neuropsicológicos, Moral

## Abstract

Multiple sclerosis (MS) is a central nervous system (CNS) disease characterized by inflammation, axonal demyelination, and neurodegeneration, which can have a strong impact on all aspects of the life of the patient. Multiple sclerosis causes motor, sensory, cerebellar, and autonomic dysfunctions, as well as cognitive and psychoemotional impairment. The most frequently compromised cognitive domains are complex attention/information processing, memory, executive and visuospatial functions. Recently, alterations have also been evidenced in complex cognitive functions, such as social cognition, moral judgment, and decision-making. Cognitive impairment is characterized by high variability and can affect work skills, social interactions, coping strategies and more generally the quality of life of patients and their families. With the use of sensitive and easy-to-administer test batteries, an increasingly accurate and early diagnosis is feasible: this allows to determine the effectiveness of possible preventive measures, to predict the future progression of the disease and to improve the quality of life of patients. There is currently limited evidence regarding the efficacy, on cognitive impairment, of disease-modifying therapies. The most promising approach, which has received strong empirical support, is cognitive rehabilitation.

## Resumo

A esclerose múltipla (EM) é uma doença do sistema nervoso central (SNC) caracterizada por inflamação, desmielinização axonal e neurodegeneração, que pode ter um forte impacto em todos os aspectos da vida dos pacientes. A EM causa disfunções motoras, sensoriais, cerebelares, autonômicas, comprometimento cognitivo e déficits psicoemocionais. Os domínios cognitivos mais frequentemente comprometidos são a atenção complexa/processamento da informação, memória, funções executivas e habilidades visuais-espaciais. Recentemente, também foram evidenciadas alterações em funções cognitivas complexas, como cognição social, julgamento moral e tomada de decisão. O comprometimento cognitivo é caracterizado por alta variabilidade e pode afetar as habilidades laborais, as interações sociais, as estratégias de enfrentamento e, de forma mais geral, a qualidade de vida dos pacientes e de seus familiares. Com o uso de baterias de testes sensíveis e fáceis de administrar, é viável um diagnóstico cada vez mais preciso e precoce: isso permite determinar a eficácia de possíveis medidas preventivas, prever a progressão futura da doença e melhorar a qualidade de vida dos pacientes. Atualmente, há evidências limitadas sobre a eficácia, no comprometimento cognitivo, de terapias modificadoras da doença. A abordagem mais promissora, que tem recebido forte apoio empírico, é a reabilitação cognitiva.

## INTRODUCTION

 Multiple Sclerosis (MS) is a chronic disease of the central nervous system (CNS) characterized by inflammation, axonal demyelination, and neurodegeneration. 1 Due to its early onset, it is the most common cause of nontraumatic neurological disability in young adults worldwide. 2 Its chronic course is characterized by relapses, remissions, and progression of disabilities that can interfere with all neurological functions. Patients with MS (pMS) present a rather heterogeneous clinical condition, characterized by motor, sensory, cerebellar, autonomic dysfunctions, cognitive impairment (CI), 3 and mood disorders. 4 For a complete review of the frequencies of various MS related disorders refer to DeLuca et al. 5

 Despite being first described by Charcot and other early authors in the mid-1800s, 6 MS-related CI has been neglected for many years, and only starting from the last quarter of the 20 ^th^ century, interest in it grew: with the use of specific and sensitive test batteries, the qualitative and quantitative characteristics of cognitive disorders have been identified and, thanks to neuroimaging techniques, correlations with the neuropathological picture have emerged. 

 Cognitive impairment can affect pMS at any stage of the disease, 4 with prevalence rates ranging from 42 to 70%. 7 Cognitive symptoms are usually hidden as opposed to more visible deficits, such as sensorimotor symptoms. Patients with MS may not be fully aware of or underestimate them with respect to emotional complaints, fatigue or pain. 8 For this reason, physicians should not rely on self-reported CI, but on the performance on objective cognitive tests. 9

 According to the most common literature in the field of MS, 7 10 11 the most frequently compromised cognitive domains are complex attention/information processing (IP), memory, executive functions (EF), and visuospatial functions. Other cognitive domains that may be altered, and which have recently been arousing great interest, are social cognition (SC), decision-making (DM) and moral judgment (MJ). 

 Like all symptoms of MS, cognitive deficits are characterized by high variability 12 and strongly impact on work skills, social interactions, and quality of life of patients. 4 An accurate and early assessment of CI is of great importance as it would allow to determine the effectiveness of possible preventive measures, predict, and monitor disease progression, 13 prevent and/or reduce the high unemployment rate associated with the disease 14 and more generally improve the quality of life of pMS. 15

Being aware that pediatric cases often show a different profile of impairment, it was decided to focus the review work on adult-onset cases. Therefore, the following topics will be covered:

- cognitive impairment in MS
- cognitive reserve and brain reserve;
- neuropsychological assessment;
- management of CI.

Considering the aim of the present work, for a complete review on the neuropathological basis of cognitive deficits related to MS as detected by neuroimaging, refer to Filippi et al. 16

## COGNITIVE IMPAIRMENT IN MS

### Complex attention/information processing

#### Attention

 Attention is a sophisticated cognitive function made up of several sub-components. A widespread clinical model divides attention into five main sub-components: focused attention; sustained attention; selective attention, alternating attention, and divided attention. 17 Attentional deficits affect ∼ 12 to 25% of pMS. 18 The attentional processes most affected in MS are the more complex ones, that is selective attention, sustained attention, and divided attention, while focused attention is often preserved. 19 Independent evaluation of this cognitive domain can be difficult. First, attention is strongly associated with IP speed (IPS) and working memory (WM): to distinguish the tasks for these different domains is complex. Second, fatigue can affect performance on attentional tasks. 20

#### Information Processing

 Information processing represents the efficiency with which neural networks transmit and integrate information. 21 The first studies on IP functioning showed that pMS needed more time than healthy controls (HCs) to determine whether a specific number was included in a series of to be remembered numbers. 22 A subsequent study, aimed at measuring both IPS and performance accuracy, suggested that when pMS were given necessary time to encode information, their performance was as that of HCs in terms of accuracy. 23 The results of these two studies show that the IP of pMS is adequate if they are given more time, so it is not the accuracy with which the information is processed, but it is IPS that is impaired. 

 Information processing speed, in addition to being the most commonly compromised cognitive function in pMS, with a percentage ranging from 40 to 70%, 7 is frequently the first to be impaired. Furthermore, IPS is important for the functionality of higher-order cognitive processes such as memory and EF. 24

 Information processing speed efficiency depends on both WM and processing speed. 19 Although both are compromised, according to some authors, IPS is the primary deficit in MS, which primarily affects information encoding, while WM impairment seems to depend on reduced IPS. 25 The study by Kouvatsou et al. evaluated the relationship between IPS and WM and found a strong correlation between IPS, central executive and episodic buffer. However, after the chronological age of the patients was entered into the statistical analyzes, IPS and central executive relationship disappeared, suggesting that this subcomponent of WM may not be directly affected by IPS, but that other factors such as age could mediate their relationship. 25

 Information processing speed is strongly related to some clinical aspects of pMS. In fact, several studies have observed that a reduced IPS correlates with an increase of motor disability, fatigue, depression, and a reduced social support quality. 24

### Memory

 Memory impairment is among the most observed cognitive deficits in MS, 4 with prevalence rates ranging from 22 to 65%. 7 20

 In most cases, long-term memory (LTM) and WM are compromised. 7 18 Recently, it has been highlighted that in MS the visual subcomponent of the visuospatial sketchpad is preserved, while the episodic buffer is the most impaired component. 26

 Regarding LTM deficits, they mainly concern explicit (declarative) memory, while implicit (nondeclarative) memory appears to be mostly preserved. 27 According to the traditional classification by Tulving et al., 28 explicit memory is divided into episodic memory and semantic memory. Of these, the first is the most compromised 29 in pMS. Episodic memory can be divided into retrospective memory (RM) and prospective memory (PM). Until now, most of the research in pMS has focused on RM. The first studies suggested that RM deficit may lie in the inability of pMS to retrieve information stored in LTM. Subsequent studies have traced the origin of problem to the initial learning difficulty: 4 pMS simply need a greater number of repetitions to acquire memory traces. Difficulty in new information acquisition could be linked to the concomitant presence of EF deficits, sensory deficits, of a slowdown in IP and of a greater susceptibility to interference. 30 Recently, it has been reported that pMS, in addition to having RM deficiencies, have some gaps in PM defined as difficulties in remembering what they intended to do. In everyday life, failure to execute a planned action (or intention) at the right time can have relevant consequences with a negative impact on the functional autonomy of patients. 31

### Visuospatial functions

 Although visuospatial functions have received less consideration, it has been reported that 25% of pMS have visual-perceptual deficits independent of the presence of primary visual deficits. 32 Visuospatial abilities impairments result in deficits in the representation and integration of images and in the spatial localization and tracking of an object. 33 A recent paper found that there is a relationship between visual IPS deficits and visual system abnormalities, defined by a reduced pMS ability to detect visual stimuli. 34

### Executive functions

 The studies conducted on EF in pMS are not easily comparable due to the high conceptual and methodological variability; in general, it is established that, in pMS, EF are altered with different degrees and frequency (20 to 80%). 35 While some authors believe that, in MS, EF deficits have a lower prevalence than memory deficits, 7 36 others declare a higher prevalence of the former. 37

 Drew et al. 37 noted that 17% of pMS have difficulty with a range of executive skills (for example, displacement, inhibition, and fluency). Using a similar approach, Cerezo Garcia et al. 35 observed a predominant influence of MS on three components of EF: cognitive flexibility, inhibition, and abstraction. This suggests that there may be a specific EF deterioration profile in MS, with better preserved planning and reasoning skills than cognitive flexibility, inhibition, and abstraction. Furthermore, pMS would seem to repeatedly propose concepts and solutions that are no longer adequate with respect to a modified situation. 38

 A relationship between EF and PM has been observed. 39 Several studies have shown that PM requires both RM and EF. Dagenais et al., 39 examining the influence of EF on pMS PM, revealed that good performance is modulated by the efficiency of EF, while RM appears to have a minimal impact on PM performance. 

 Recently, it has been found that EF deficits can be largely explained by a loss of general intelligence. 40 In fact, both cognitive functions are supported by the prefrontal cortex (PFC) and the EF deficits have been found to be related to lesions of the frontosubcortical tracts. 

 Leavitt and colleagues believe that EF deficits may be partly explained by IPS deficits: evaluating and comparing pMS with HC, they found that the former had worse scores than HCs in EF and IPS, but that the differences diminished when more time is provided. 41

 In a 2017 study, a relationship between EF and coping 42 was demonstrated: active coping strategies (such as problem solving) require prediction and perspective taking. The alteration of these abilities would lead pMS to use maladaptive and less cognitively demanding coping strategies (for example, acceptance or avoidance), with negative consequences on quality of life. 

### Language

 In MS, language has been poorly studied, and while some researches have shown largely preserved linguistic functionality, 7 more recent studies have reported speech disturbances in 20% of relapsing-remitting pMS (RRMS) and in 58% of secondary-progressive pMS (SPMS). 43 Some researchers argue that pMS experience subtle language deficits not easily detectable, 44 such as the difficulty in finding words. 45 Indeed, several papers suggest that individuals with MS show deficits in pragmatics 46 and in morphosyntactic production. 47 48 Typically, verbal fluency (VF) is the only language-related measure included in the batteries used to assess cognitive deficits in pMS. A standard VF test is aimed at evaluating phonemic and/or semantic fluency. Often, difficulties in VF are attributed to EF deficits rather than language skills. 4 In general, as phonemic characteristics are cognitively more demanding, pMS have better performance in the semantic than in the phonemic fluency task. Indeed, phonemic fluency is more based on cognitive control and EF, while semantic fluency is dependent on semantic knowledge 49 and episodic memory. 50 This dissociation is supported by the fact that phonemic fluency is associated with white matter dorsal (occipito-parietal) tract integrity, while semantic fluency is associated with ventral (occipito-temporal) tract integrity. 51 Despite this, research conducted with healthy individuals indicates that both EF, IPS, and language-specific abilities contribute to VF. Furthermore, a recent study has shown that both vocabulary and IPS can predict phonemic fluency; otherwise, semantic fluency is uniquely related to vocabulary. These results suggest that VF deficits in pMS are the consequence of a reduction in both language skills and IPS. 44

 In MS, aphasia is a very rare clinical manifestation, with a percentage ranging from 0.7 to 3%. 52 Among the various forms of aphasia, Broca's aphasia appears to be the most frequent in pMS, followed by mixed transcortical aphasia. 53 Although most cases of aphasia are induced by cortical lesions, unusually extensive subcortical white matter lesions have been reported in MS. In this regard, extensive plaques (> 5-cm) have been identified in language subcortical areas of pMS. As an explanation for subcortical injury-induced aphasia, the concept of diaschisis has been proposed. According to this, lesions of white matter tracts, anatomically related to cortical language centers, could produce aphasias that are sometimes difficult to distinguish from aphasias associated with cortical lesions. 54

### Cognitive impairment according to clinical phenotype

 Cognitive impairment occurs in all MS phenotypes, affecting between 20 and 25% of patients with clinically isolated syndrome (CIS) and radiologically isolated syndrome (RIS); 30 to 45% of RRMS and 75% of patients with SPMS. The prevalence of CI in primary-progressive MS (PPMS) is very variable. Clinically isolated syndrome and RRMS show similar cognitive profiles with significant involvement of IPS, EF, and verbal and visuospatial memory. However, a higher overall CI index was observed in RRMS patients than in those with CIS. A CI profile like that of RRMS was observed in RIS patients, with involvement of IPS and memory. 55 Progressive forms have a relatively similar cognitive profile, with a prevalent involvement of EF and memory. 12 In a study conducted by Dackovic et al., 56 patients with PPMS and SPMS were found to be more frequently compromised than those with CIS and RRMS in all cognitive tests evaluated by the Brief Repeatable Battery of Neuropsychological Tests (BRBNT). 10 Until now, pMS have always been classified as cognitively preserved or cognitively impaired. With the aim of eliminating this too reductive dichotomous classification, a group of researchers 57 managed to identify 5 cognitive phenotypes: preserved cognition; mild involvement of verbal memory and semantic fluency; mild multidomain involvement; severe executive/attentional involvement; and severe multidomain involvement. Furthermore, in support of Dackovic's work, 56 they found that progressive phenotypes are those with more prevalent CI. 

### Cognitive impairment and mood disorders

 Patients with MS are often diagnosed with mood disorders such as: major depressive disorder, bipolar disorder, anxiety disorders, euphoria, and pseudobulbar syndrome. Among these, depressive and anxiety disorders were found to be correlated with CI. The most common depressive symptoms in MS include irritability, discouragement, concentration problems, fatigue, insomnia, and poor appetite; while guilt, low self-esteem, and social withdrawal are less frequent than in the general population. 58 These symptoms affect 50% of pMS. Prevalence estimates are generally 2-3 times higher than those of the general population. 59 Several studies have suggested that depression, in pMS, is associated with WM, EF and IPS deficits. In contrast to the extensive literature on depression, in pMS, anxiety disorders have been the subject of fewer studies. The prevalence of anxiety disorders in MS is estimated to be between 13 and 31.7%, 60 and it is 3 times higher in the latter than in the general population. 61 Generalized anxiety disorder appears to be the most common form, with 18.6% of patients meeting the criteria. This is followed by panic disorder (10%) and obsessive-compulsive disorder (8.6%). 62 Several studies have shown the existence of an association between anxiety and attention, WM, IPS, immediate and delayed visual memory, VF, and verbal memory deficits. 63 64 65 66

### Cognitive impairment and other factors

 Recently, factors directly associated with the pathophysiological mechanisms of MS have been identified, which can contribute to the development of CI. Noteworthy are sleep disturbances and fatigue. About 50% of pMS suffer from sleep disorders. 67 Studies examining the relationships between sleep disorders and CI have produced discrepant association patterns derived from the use of different sleep measurement tools: using objective measures (polysomnography and actigraphy), sleep was found to correlate with attention and IPS. Subjective measures (of self-reported insomnia through the Pittsburgh Sleep Quality Index, Insomnia Severity Index) were found to be predictive of self-reported cognitive deficits; however, these associations were mediated by depression and fatigue, suggesting that these symptoms may contribute to the CI perceived by pMS. Unlike objective measures, self-reported sleep was a poor indicator of CI detected by standardized batteries. 68 One of the most commonly complained and most disabling symptoms is fatigue, which affects up to 75 to 90% of pMS. 69 Unlike some research, 70 71 recent studies have highlighted the existence of a strong association between fatigue, attention 72 and IPS. 73 Another variable that can influence CI is hypothyroidism: a syndrome caused by insufficient secretion of thyroid hormone. Thyroid hormones have a vital impact on normal brain development and function, as well as cognition. Clinical psychology studies have shown that the attention, memory and spatial capacity of patients with hypothyroidism are significantly reduced, while the index of depression and anxiety is increased. 74 Also, hyperhomocysteinemia, vitamin B12 and folate deficiency have been linked to CI in MS. 75

### Social cognition

 Social cognition is a multidimensional construct including a wide range of neurocognitive processes that allow humans to perceive and make inferences about mental states, understand the behavior of others, interact adequately with other people, and adaptively orient their behaviors towards appropriate goals. 76 77 Social perception, social understanding and social DM are the basilar processes of SC. The first refers to the perception of emotions through prosody or facial expressions, the second refers to affective empathy, that is, the ability to experience and interpret others' feelings, while the third process concerns the theory of mind (ToM) or mentalization, defined as the ability to decode and interpret the mental states of others and use them to make inferences and predict their behaviors. 76 78 These skills are vital for the development of complex social interactions and can impact employment, relationships with family, friends, and healthcare professionals. 79

 A total of 20 to 40% of pMS have a SC deterioration, sometimes evident from the early stages of the disease. 80 In pMS, SC disorders generally do not correlate with disease course and duration, degree of disability, or relapse rate. 80 Otherwise, a correlation between SC and fatigue was found, probably due to common underlying neural networks. 81

 Recently, numerous studies investigated the relationship between SC and CI in pMS leading to inconsistent results. While, on the one hand, significant correlations were found between SC and IPS, WM, reasoning, and problem-solving deficits, on the other hand, no association was found among these variables. 77

 Deficits in facial emotional recognition (FER) and ToM are the most frequent in pMS and, therefore, also the most investigated. 76 Theory of mind is a multifaceted construct that can be further divided into cognitive and affective components. 82 A recent meta-analysis 77 conducted on 1,708 pMS and 1,518 HCs showed that compared to the latter, pMS (RRMS, PPMS, SPMS) showed alterations in the overall ToM, of its two subcomponents, and FER. Usually, no differences in impairment levels were observed between MS phenotypes. In contrast, Argento et al. 83 demonstrated the presence of specific patterns of impairment of SC skills by phenotype: both RRMS and SPMS performed poorly on the Reading the Mind in the Eyes test compared with HCs, but only SPMS patients reached a statistically significant difference. 83 Individuals with MS are not only less accurate in recognizing basic emotions than healthy individuals, but they have longer reaction times. 84

 Regarding empathy, while some studies have highlighted subtle difficulties in pMS, 84 recent research found no difference in empathy between pMS and the healthy population. 79 In pMS, a correlation was observed between higher empathy levels and a higher education level, better verbal learning, fewer depressive symptoms, greater extroversion, higher levels of agreeableness and conscientiousness, and better occupational functioning. 79

 Currently, great importance for the empathy's modulation has been given to alexithymia. 85 Indeed, a higher incidence of alexithymia in pMS was associated with lower levels of empathy and potentially impaired moral cognition, even in the early stages of the disease. 86 Although it can affect ∼ 10% of the general population, the prevalence of alexithymia in pMS can reach 53%, 87 becoming a variable of great interest when evaluating SC. 

### Moral judgment

 The set of habits and values that guide social conduct in a given cultural group is called MJ, and is measured through tasks that involve moral dilemmas, that is, situations in which an agent cannot meet all applicable moral requirements. 88 Moral judgement deficits can be deleterious to both pMS and their social circle; despite the emerging literature on socioemotional skills, moral cognition is still poorly studied in MS. 89 90 Three important aspects of MJ are moral acceptability/admissibility related to an acted-out behavior (that is, morally acceptable, or unacceptable), emotional valence (evaluation of the experience as pleasant or unpleasant) and emotional arousal (state of arousal or calm). 91

 Preliminary evidence 89 90 demonstrated a reduced valuation capacity of moral acceptability, higher levels of emotional reactivity and an egocentric projection of the moral problem. These results led to the hypothesis that, in MS, there is a shift from a MJ "utilitarian model", based on a careful cost-benefit analysis of the specific moral situation, towards a "nonutilitarian moral model" 89 driven by an instinctive emotional aversion to harm other people. These data were partly supported by a recent study 92 : the authors observed a reduced sensitivity to moral admissibility in RRMS patients compared with HCs; on the other hand, they found reduced emotional reactivity levels suggesting an alteration in the emotional reaction rather than in the evaluation of moral dilemmas per se. Emotional detachment can be explained according to two different hypotheses: the presence of high levels of alexithymia impairing emotional reactivity (reduced emotional arousal) during DM in moral dilemmas; 89 the fact that pMS implement coping strategies based on emotional detachment to maintain a good quality of life and obtain a better adaptation to their social context. 93

### Decision-making

 Decision-making is commonly perceived as a highly rational and conscious process that allows to comparing arguments for or against a specific behavioral choice. 94 Decisione-making is organized according to a well-defined basic structure. input-process-output-feedback. Input refers to the presentation of individual stimuli, each of which is capable of providing a rewarding or punishing response; the process allows you to evaluate the stimuli in order to choose one according to your preferences; the output refers to the action implemented in response to the chosen stimulus; feedback refers to the assessment and experience made by the subject following the action taken. It is evident that DM is guided by both cognitive and emotional components associated with a vast cortico-subcortical network. 95 A theory supporting the significant influence of emotions on DM performance is that of the somatic marker, developed by Damasio. 96 This theory states that, when faced with a decision, a first selection of the choices is made by balancing the positive and negative somatic markers. Accordingly, the choice would be conditioned by the somatic and emotional responses experienced by the subject, sometimes unconsciously. 96 In this regard, two studies that measured emotional reactivity using the reactivity of skin conductance (RSC) have given conflicting results. In one study, it was found that pMS, compared with HCs, had a reduced RSC in DM performance under ambiguity (when neither the outcome nor the probability of specific results is known), which, however, was preserved in the DM under risk (when the probabilities for possible outcome scenarios are given but the outcome is not). 97 In the second study, however, the pMS showed reduced expression of only negative emotions (disappointment and regret) in the face of negative outcomes of their choices. 98

 In the healthy population, good memory capacity predicts adaptive DM in a wide range of cognitive tasks 99 and real-life contexts. 100 In pMS, the worst decisions were found to correlate with IPS, EF, and memory deficits. 101 Regarding memory, several studies have highlighted how WM deficits are able to predict resistance to framing (influence on DM of the way information is presented) and the ability to follow decision rules, 101 suggesting an involvement of WM in supporting the most cognitively demanding decisions. Semantic memory turned out to predict an over and/or underestimation of risk perception and consistency in the application of decision rules: this suggests that DM tasks (ranging from understanding complex instructions to judging the probability of events) require the recovery of knowledge previously learned and stored in LTM. 101 A recent review 95 that analyzed 12 studies found that 64.7% of pMS exhibit a DM performance reduction: 67% of patients have an alteration in under risk DM performance and 64% in under ambiguity DM. Furthermore, all the included studies indicated a tendency for pMS to seek risk: this could be explained by reward hypersensitivity or a reduced ability to assess immediate gain versus long-term outcome, a so-called "myopia for the future." 95 Recently, it has been shown that although RRMS patients are able to collect as much information as HCs before deciding, they are twice as likely to make irrational decisions, that is, against gathered evidence. Furthermore, in line with previous studies, it has been confirmed that RRMS patients are more influenced by the way information is presented (framing effect) than HC. Overall, these results push towards greater caution in communicating with pMS, especially regarding medical information. 102

## COGNITIVE RESERVE AND BRAIN RESERVE

 The theory of cognitive reserve postulates that life experiences (education, work, hobbies) interacting with hereditary/genetic and environmental factors can influence the efficiency and flexibility of brain networks, allowing individuals to better cope with neurodegenerative processes at the basis of aging and brain diseases. 103 Specifically, increased intellectual enrichment mitigates the negative effect of the disease burden of MS on cognitive status. 104 Differently, the concept of brain reserve refers to the structural characteristics of the brain at any given moment. It can protect individuals from age-related or disease-related brain changes by influencing the critical threshold after which cognitive decline emerges. 103 For example, people with bigger maximal lifetime brain growth (MLBG; estimated based on head size or intracranial volume) can bear a more severe disease burden without developing CI. These subjects, compared with those with reduced MLBG, may lose more brain volume before exceeding the critical threshold assigned to CI. These considerations can help to identify patients at greater risk of CI and therefore to intervene early with specific rehabilitation interventions. 104

## NEUROPSYCHOLOGICAL ASSESSMENT

 Tests commonly used to screen for cognitive deficits in dementia, such as the Mini-Mental State Examination or the Montreal Cognitive Assessment, which primarily assess cortical functions, are not sufficiently sensitive and specific to assess the domains typically affected in MS. Neuropsychological Screening Battery for MS (NSBMS) 10 is one of the first neuropsychological test batteries used for the assessment of cognitive deficits in MS, developed by neuroscientists of the Cognitive Function Study Group. This battery included the Selective Reminding Test (SRT), the 7/24 Spatial Recall Test (SPART), the Paced Auditory Serial Addition Test (PASAT) and the Word List Generation Test (WGLT). Subsequently, the same group proposed the applicability of the BRBNT, integrating the Symbol Digit Modality Test (SDMT) and using the SPART 10/36 instead of 7/24 version. 15 Although the BRBNT is considered one of the most valid neuropsychological test batteries for CI evaluation in pMS, some uncertainties have been raised: the first concerns the absence of sufficient data regarding psychometric properties for test-retest evaluations and for the use of parallel forms; the second is that it does not cover all the cognitive domains deficient in MS. 105

 To overcome these limitations, a conference was held with 16 clinical psychologists and neuropsychologists to recommend a minimum set of neuropsychological tests to be included in a routine evaluation. A new battery of tests called Minimal Assessment of Cognitive Functioning in Multiple Sclerosis (MACFIMS) 11 emerged, in which the 10/36 SPART was replaced with the Brief Visuospatial Memory Test Revised (BVMT-R) and the SRT has been replaced with the California Verbal Learning Test-Second Edition (CVLT-II). In addition, two new tests have been added: Judgment of Line Orientation (BJLO) and Delis-Kaplan Executive Function System Sorting Test (D-KEFS ST). Although this battery has considerable validity as a tool for the neuropsychological assessment of pMS, unfortunately the long time required for its administration and interpretation, and the high costs due to the need for adequately trained personnel, make it difficult to use outside specialized centers. To overcome the aforementioned problems, in 2010 an expert panel recommended the use of the Brief International Cognitive Assessment for Multiple Sclerosis (BICAMS). 106 This battery includes the SDMT, the first five trials of the CVLT-II and the first three trials of the BVMT-R. 105 A reliable and sensitive endpoint to be used to determine the efficacy of disease-modifying drugs in improving cognitive functioning in pMS is the Multiple Sclerosis Cognition Assessment Battery (MS-COG). 107 This battery of tests is composed of SDMT, PASAT 3 'and 2', SRT and BVMT-R. 105 Table 1 shows the main neuropsychological batteries used for the evaluation of pMS, with the respective tests and cognitive domains investigated. 

**Table 1:** Table showing the main neuropsychological batteries used for evaluation in MS, the tests that compose them, and the cognitive domains under investigation

Battery	Test	Cognitive Domain
	SRT	Verbal learning and memory
**NSBMS**	7/24 SPART	Visuospatial learning and memory
	WGLT	Verbal fluency and word retrieval
	PASAT	Working memory and processing speed
**BRBNT**	SRT 10/36 SPART SDMT WGLT PASAT	Verbal learning and memory Visuospatial learning and memory Processing speed Verbal fluency and word retrieval Working memory and processing speed
**MACFIMS**	PASAT	Working memory and/or processing speed
	CVLT-II	Verbal learning and memory
	BVMT-R	Visuospatial learning and memory
	SDMT	Processing speed
	BJLO	Visuospatial processing
	COWAT D-KEFS ST	Verbal fluency or word retrieval Executive functioning and problem solving
**BICAMS**	SDMT BVMT-R CVLT-II	Processing speed Visuospatial learning and memory Verbal learning and memory
**MS-COG**	PASAT	Working memory and processing speed
	SRT	Verbal learning and memory
	BVMT-R	Visuospatial learning and memory

Abbreviations: 10/36 SPART, 10/36 Spatial Recall Test; 7/24 SPART, 7/24 Spatial Recall Test; BICAMS, The Brief International Cognitive Assessment for Multiple Sclerosis; BJLO, Judgment of Line Orientation; BRBNT, The Brief Repeatable Battery of Neuropsychological Tests; BVMT-R, Brief Visuospatial Memory Test Revised; CVLT-II, California Verbal Learning Test-Second Edition; D-KEFS ST, Delis-Kaplan Executive Function System Sorting Test; MACFIMS, The Minimal Assessment of Cognitive Functioning in Multiple Sclerosis; MS-COG, Multiple Sclerosis Cognition Assessment Battery; NSBMS, The Neuropsychological Screening Battery for MS; PASAT, Paced Auditory Serial Addition Test; SDMT, Symbol Digit Modality Test; SRT, Selective Reminding Test; WGLT, Word List Generation Test.

 In case of limited available time, long test batteries can be substituted by more targeted tests. The single most sensitive and specific test to identify CI in pMS is the SDMT. 15 Furthermore, the SDMT is a significant marker of active disease, as it is the only test that provides results consistently associated with isolated cognitive relapses (ICR). 12 While transient cognitive disturbances are described in association with other symptomatic neurological deficits during disease activity, ICR is characterized by a significant transient cognitive decline in objective neuropsychological performance, with no clinical or subjective evidence of other associated new neurological signs and symptoms to the activity of brain disease defined by the increase of gadolinium on magnetic resonance. 108

 Due to the physical disabilities of pMS, the oral versions are preferred. SDMT is relatively free from practice effects, so it is suitable for serial assessments. Furthermore, it is highly predictive of the future cognitive decline and unemployment status of pMS. 15

 Recently, van Oirschot et al. 109 validated a variant of SDMT (sSDMT; Orikami Digital Health Products) that can be used on smartphones via the MS sherpa application. Obviously, interest in cognitive tests with a digital interface is increasing. These, in fact, would not only allow an automated assessment of cognitive functions, but would reduce the need for qualified professionals. 12 For a review of computerized tests, refer to the work of Wojcik et al. 110 Other tests believed to be effective in distinguishing pMS from HCs are the memory test: CVLT, the BVMT and the Rey Auditory Verbal Learning Test (RAVLT). 12

In standard clinical practice, usually, the neuropsychological assessment conducted using objective measures is completed by the use of self-assessment questionnaires and semi-structured interviews that allow to have a more complete view of the impact of CI on the daily functioning of patients.

 Among the subjective measures, stands out the Neuropsychological Questionnaire on Multiple Sclerosis, 111 which provides useful information on the patient's perception of cognitive difficulties. 105

## MANAGEMENT OF COGNITIVE IMPAIRMENT

### Pharmacological treatment

 Evidence supporting a positive influence of disease-modifying therapies (DMTs) on MS-related CI is limited, and to date no drugs have been approved. 112 113 No class I evidence was detected for these types of drugs, and class II investigations showed small, non-significant or negligible effects. 112 On the other hand, class III and IV observational studies have produced positive results. Despite this, due to a multitude of methodological limitations, their results cannot be generalized. 112 In contrast to DMTs studies, those conducted on symptomatic therapies produced stronger treatment effect sizes. However, considering the data emerged so far, it is not yet possible to draw conclusions regarding their effectiveness on CI. 112 114

### Rehabilitation

 Cognitive rehabilitation (CR) consists of a series of behavioral treatments aimed at helping patients improve their cognitive functions and daily living activities. Generally, there are two different approaches to CR: restorative and compensatory. Restorative CR (rCR) aims to strengthen and restore cognitive abilities using repetitive cognitive exercises based on computer-assisted paradigms. Differently, compensatory CR (cCR) aims to compensate cognitive difficulties of patients through the use of various internal (for example, visualization) and external (for example, reminder) 115 strategies. A rehabilitation program supported by a randomized controlled trial (RCT) is the BrainHQ, composed of 15 specific exercises for EF, IPS, attention, and WM. Attention Processing Training (APT) was found to be effective in the treatment of attention deficits, while the Cognitive Training Kit (COGNI-TRAcK) was found to be optimal in the rehabilitation of WM. 116

 One of the most widely used computer programs for pMS CR is RehaCom, a software consisting of several specific modules for different cognitive domains (attention, concentration, memory, perception), activities of daily living and much more. It can be used both on single domains and on multiple cognitive processes simultaneously. Its effectiveness has been supported by several RTCs. 115 Excellent results have been observed from the use of REHACOP, an integrative neuropsychological rehabilitation program that trains various cognitive functions including attention, IPS, learning, memory, language, EF, and SC. 117

 Regarding cCR, two programs that have been shown to significantly improve learning and memory of pMS are the Story Memory Technique (KF-mSMT) and the Kessler Foundation Strategy Based Techniques to Enhance Memory (KF- STEM). 115

 Currently, several studies have documented the benefits induced by exercise on the cognitive functions of healthy and elderly adults with CI and in patients with various neuropsychiatric diseases. Despite this, due to conflicting results, a recent 3-level meta-analysis underline the lack of sufficient evidence in favor of motor rehabilitation as a treatment for pMS cognitive deficits. 118

In conclusion, although starting only in the last quarter of the last century, interest and knowledge about CI in MS grew, research and clinical experience have revealed a strong influence of cognitive deficits on many aspects of the daily life of patients and their families. For these reasons, the assessment of cognitive functioning should be standard clinical practice aimed at ensuring early diagnosis, preventing and/or predicting future disease progression and implementing adequate treatment supported by scientific evidence. If time and resources were available, it would be desirable to prioritize the use of test batteries, rather than single screening tools, accompanied by self-assessment questionnaires and semi-structured interviews that allow a more complete view of the impact of CI on the daily functioning of patients. This type of neuropsychological assessment would allow the clinician not only to investigate all cognitive domains and identify those specifically compromised, but also to develop targeted and specific rehabilitation treatments that take into account the challenges and daily needs of each patient. Furthermore, the strong associations between CI, mood disorders and factors directly related to the pathophysiology of MS should be considered. Thus, specific treatments could be developed for these variables that indirectly affect cognition.

## References

[1] Halabchi F Alizadeh Z Sahraian M A Abolhasani M Exercise prescription for patients with multiple sclerosis; potential benefits and practical recommendations BMC Neurol 2017 17 01 185 10.1186/s12883-017-0960-9 28915856 PMC5602953

[2] Browne P Chandraratna D Angood C Atlas of Multiple Sclerosis 2013: A growing global problem with widespread inequity Neurology 2014 83 11 1022 1024 10.1212/WNL.0000000000000768 25200713 PMC4162299

[3] Maggio M G Russo M Cuzzola M F Virtual reality in multiple sclerosis rehabilitation: A review on cognitive and motor outcomes J Clin Neurosci 2019 65 65 106 111 10.1016/j.jocn.2019.03.017 30898488

[4] Macías Islas MÁ Ciampi E Assessment and Impact of Cognitive Impairment in Multiple Sclerosis: An Overview Biomedicines 2019 7 01 22 30893874 10.3390/biomedicines7010022 PMC6466345

[5] DeLuca J Nocentini U Neuropsychological, medical and rehabilitative management of persons with multiple sclerosis NeuroRehabilitation 2011 29 03 197 219 10.3233/NRE-2011-0695 22142753

[6] Charcot J M Lectures on the diseases of the nervous system delivered at la Salpetriere, London:New Sydenham Society;1877

[7] Chiaravalloti N D DeLuca J Cognitive impairment in multiple sclerosis Lancet Neurol 2008 7 12 1139 1151 10.1016/S1474-4422(08)70259 19007738

[8] Kinsinger S W Lattie E Mohr D C Relationship between depression, fatigue, subjective cognitive impairment, and objective neuropsychological functioning in patients with multiple sclerosis Neuropsychology 2010 24 05 573 580 10.1037/a0019222 20804245 PMC2933087

[9] Goverover Y Chiaravalloti N DeLuca J The relationship between self-awareness of neurobehavioral symptoms, cognitive functioning, and emotional symptoms in multiple sclerosis Mult Scler 2005 11 02 203 212 10.1191/1352458505ms1153oa 15794396

[10] Rao S M Leo G J Bernardin L Unverzagt F Cognitive dysfunction in multiple sclerosis. I. Frequency, patterns, and prediction Neurology 1991 41 05 685 691 10.1212/wnl.41.5.685 2027484

[11] Benedict R H Fischer J S Archibald C J Minimal neuropsychological assessment of MS patients: a consensus approach Clin Neuropsychol 2002 16 03 381 397 10.1076/clin.16.3.381.13859 12607150

[12] Benedict R HB Amato M P DeLuca J Geurts J JG Cognitive impairment in multiple sclerosis: clinical management, MRI, and therapeutic avenues Lancet Neurol 2020 19 10 860 871 10.1016/S1474-4422(20)30277-5 32949546 PMC10011205

[13] Moccia M Lanzillo R Palladino R Cognitive impairment at diagnosis predicts 10-year multiple sclerosis progression Mult Scler 2016 22 05 659 667 10.1177/1352458515599075 26362896

[14] Dorstyn D S Roberts R M Murphy G Haub R Employment and multiple sclerosis: A meta-analytic review of psychological correlates J Health Psychol 2019 24 01 38 51 10.1177/1359105317691587 28810436

[15] Oset M Stasiolek M Matysiak M Cognitive Dysfunction in the Early Stages of Multiple Sclerosis-How Much and How Important? Curr Neurol Neurosci Rep 2020 20 07 22 10.1007/s11910-020-01045-3 32444997 PMC7244611

[16] Filippi M Rocca M A Benedict R H The contribution of MRI in assessing cognitive impairment in multiple sclerosis Neurology 2010 75 23 2121 2128 10.1212/WNL.0b013e318200d768 21135387 PMC3385423

[17] Nocentini U Romano S Caltagirone C. I deficit cognitivi nella sclerosi multipla. in Nocentini U, Caltagirone C, Tedeschi G. I disturbi neuropsichiatrici nella sclerosi multipla. Milano: Springer; 2011. 123-144. DOI:https://doi.org/10.1007/978-88-470-1711-5

[18] Guimarães J Sá M J Cognitive dysfunction in multiple sclerosis Front Neurol 2012 3 03 74 10.3389/fneur.2012.00074 22654782 PMC3359427

[19] Oreja-Guevara C Ayuso Blanco T Brieva Ruiz L Hernández Pérez MÁ Meca-Lallana V Ramió-Torrentà L Cognitive Dysfunctions and Assessments in Multiple Sclerosis Front Neurol 2019 10 10 581 10.3389/fneur.2019.00581 31214113 PMC6558141

[20] Grzegorski T Losy J Cognitive impairment in multiple sclerosis - a review of current knowledge and recent research Rev Neurosci 2017 28 08 845 860 10.1515/revneuro-2017-0011 28787275

[21] DeLuca J Kalmar J H. Information Processing Speed in Clinical Populations. New York: Psychology Press; 2007 DOI:10.4324/9780203783054

[22] Rao S M St Aubin-Faubert P Leo G J Information processing speed in patients with multiple sclerosis J Clin Exp Neuropsychol 1989 11 04 471 477 10.1080/01688638908400907 2760182

[23] Demaree H A DeLuca J Gaudino E A Diamond B J Speed of information processing as a key deficit in multiple sclerosis: implications for rehabilitation J Neurol Neurosurg Psychiatry 1999 67 05 661 663 10.1136/jnnp.67.5.661 10519876 PMC1736611

[24] Eizaguirre M B Vanotti S Merino Á The Role of Information Processing Speed in Clinical and Social Support Variables of Patients with Multiple Sclerosis J Clin Neurol 2018 14 04 472 477 10.3988/jcn.2018.14.4.472 30198225 PMC6172515

[25] Kouvatsou Z Masoura E Kimiskidis V Working Memory Deficits in Multiple Sclerosis: An Overview of the Findings Front Psychol 2022 13 13 866885 10.3389/fpsyg.2022.866885 35572228 PMC9097870

[26] Kouvatsou Z Masoura E Kiosseoglou G Kimiskidis V K Working memory profiles of patients with multiple sclerosis: Where does the impairment lie? J Clin Exp Neuropsychol 2019 41 08 832 844 10.1080/13803395.2019.1626805 31204607

[27] González Torre J A Cruz-Gómez ÁJ Belenguer A Sanchis-Segura C Ávila C Forn C Hippocampal dysfunction is associated with memory impairment in multiple sclerosis: A volumetric and functional connectivity study Mult Scler 2017 23 14 1854 1863 10.1177/1352458516688349 28086035

[28] Tulving E Schacter D L Priming and human memory systems Science 1990 2474940301 306 10.1126/science.2296719 2296719

[29] TuSCIMS Study Group Amato M P Portaccio E Goretti B Relevance of cognitive deterioration in early relapsing-remitting MS: a 3-year follow-up study Mult Scler 2010 16 12 1474 1482 10.1177/1352458510380089 20729256

[30] D'Amico E Leone C Hayrettin T Patti F Can we define a rehabilitation strategy for cognitive impairment in progressive multiple sclerosis? A critical appraisal Mult Scler 2016 22 05 581 589 10.1177/1352458516632066 26920381

[31] Rouleau I Dagenais E Tremblay A Prospective memory impairment in multiple sclerosis: a review Clin Neuropsychol 2018 32 05 922 936 10.1080/13854046.2017.1361473 28774220

[32] Poole J L Nakamoto T McNulty T Dexterity, visual perception, and activities of daily living in persons with multiple sclerosis Occup Ther Health Care 2010 24 02 159 170 10.3109/07380571003681202 23898901

[33] Marasescu R Cerezo Garcia M Aladro Benito Y Impairment of visuospatial/visuoconstructional skills in multiple sclerosis patients: the correlation with regional lesion load and subcortical atrophy Neurologia 2016 31 03 169 175 10.1016/j.nrl.2015.06.003 26342250

[34] Lopes Costa S Gonçalves O F DeLuca J Chiaravalloti N Chakravarthi R Almeida J The Temporal Dynamics of Visual Processing in Multiple Sclerosis Appl Neuropsychol Adult 2016 23 02 133 140 10.1080/23279095.2015.1020157 26508328

[35] Cerezo García M Martín Plasencia P Aladro Benito Y Alteration profile of executive functions in multiple sclerosis Acta Neurol Scand 2015 131 05 313 320 10.1111/ane.12345 25659411

[36] Sehanovic A Smajlovic D Tupkovic E Cognitive Disorders in Patients with Multiple Sclerosis Mater Sociomed 2020 32 03 191 195 10.5455/msm.2020.32.191-195 33424448 PMC7780783

[37] Drew M Tippett L J Starkey N J Isler R B Executive dysfunction and cognitive impairment in a large community-based sample with Multiple Sclerosis from New Zealand: a descriptive study Arch Clin Neuropsychol 2008 23 01 1 19 10.1016/j.acn.2007.09.005 17981008

[38] Birnboim S Miller A Cognitive strategies application of multiple sclerosis patients Mult Scler 2004 10 01 67 73 10.1191/1352458504ms980oa 14760955

[39] Dagenais E Rouleau I Tremblay A Prospective memory in multiple sclerosis: The impact of cue distinctiveness and executive functioning Brain Cogn 2016 109 109 66 74 10.1016/j.bandc.2016.07.011 27643953

[40] Goitia B Bruno D Abrevaya S The relationship between executive functions and fluid intelligence in multiple sclerosis PLoS One 2020 15 04 e0231868 10.1371/journal.pone.0231868 32320404 PMC7176096

[41] Leavitt V M Wylie G Krch D Chiaravalloti N DeLuca J Sumowski J F Does slowed processing speed account for executive deficits in multiple sclerosis? Evidence from neuropsychological performance and structural neuroimaging Rehabil Psychol 2014 59 04 422 428 10.1037/a0037517 25133903

[42] Grech L B Kiropoulos L A Kirby K M Butler E Paine M Hester R Executive function is an important consideration for coping strategy use in people with multiple sclerosis J Clin Exp Neuropsychol 2017 39 08 817 831 10.1080/13803395.2016.1270907 28092209

[43] Ntoskou K Messinis L Nasios G Cognitive and language deficits in multiple sclerosis: comparison of relapsing remitting and secondary progressive subtypes Open Neurol J 2018 12 12 19 30 10.2174/1874205X01812010019 29576812 PMC5850485

[44] Lebkuecher A L Chiaravalloti N D Strober L B The role of language ability in verbal fluency of individuals with multiple sclerosis Mult Scler Relat Disord 2021 50 50 102846 10.1016/j.msard.2021.102846 33626431

[45] Brandstadter R Fabian M Leavitt V M Word-finding difficulty is a prevalent disease-related deficit in early multiple sclerosis Mult Scler 2020 26 13 1752 1764 10.1177/1352458519881760 31741430 PMC7234894

[46] Chanial C Basaglia-Pappas S Jacqueline S Assessment of implicit language and theory of mind in multiple sclerosis Ann Phys Rehabil Med 2020 63 02 111 115 10.1016/j.rehab.2019.08.005 31586684

[47] Fyndanis V Messinis L Nasios G Impaired Verb-Related Morphosyntactic Production in Multiple Sclerosis: Evidence From Greek Front Psychol 2020 11 11 2051 10.3389/fpsyg.2020.02051 32973621 PMC7481395

[48] Sonkaya A R Bayazit Z Z Language Aspects of Patients with Multiple Sclerosis EJMI 2018 2 03 133 138 10.14744/ejmi.2018.96158

[49] Friesen D C Luo L Luk G Bialystok E Proficiency and Control in Verbal Fluency Performance across the Lifespan for Monolinguals and Bilinguals Lang Cogn Neurosci 2015 30 03 238 250 10.1080/23273798.2014.918630 25642427 PMC4309987

[50] Delgado-Álvarez A Matias-Guiu J A Delgado-Alonso C Cognitive Processes Underlying Verbal Fluency in Multiple Sclerosis Front Neurol 2021 11 11 629183 10.3389/fneur.2020.629183 33551984 PMC7859643

[51] Blecher T Miron S Schneider G G Achiron A Ben-Shachar M Association Between White Matter Microstructure and Verbal Fluency in Patients With Multiple Sclerosis Front Psychol 2019 10 10 1607 10.3389/fpsyg.2019.01607 31379663 PMC6657651

[52] Naro L D Spinello R S Cantone M Rehabilitative treatment in a case of aphasia as onset of multiple sclerosis Neurol Sci 2021 42 09 3919 3921 10.1007/s10072-021-05364-2 34125324

[53] Demirkiran M Ozeren A Sönmezler A Bozdemir H Crossed aphasia in multiple sclerosis Mult Scler 2006 12 01 116 119 10.1191/135248506ms1255cr 16459730

[54] Devere T R Trotter J L Cross A H Acute aphasia in multiple sclerosis Arch Neurol 2000 57 08 1207 1209 10.1001/archneur.57.8.1207 10927803

[55] Brochet B Ruet A Cognitive Impairment in Multiple Sclerosis With Regards to Disease Duration and Clinical Phenotypes Front Neurol 2019 10 10 261 10.3389/fneur.2019.00261 30949122 PMC6435517

[56] Dackovic J Pekmezovic T Mesaros S The Rao's Brief Repeatable Battery in the study of cognition in different multiple sclerosis phenotypes: application of normative data in a Serbian population Neurol Sci 2016 37 09 1475 1481 10.1007/s10072-016-2610-1 27207679

[57] De Meo E Portaccio E Giorgio A Identifying the Distinct Cognitive Phenotypes in Multiple Sclerosis JAMA Neurol 2021 78 04 414 425 10.1001/jamaneurol.2020.4920 33393981 PMC7783596

[58] Silveira C Guedes R Maia D Curral R Coelho R Neuropsychiatric Symptoms of Multiple Sclerosis: State of the Art Psychiatry Investig 2019 16 12 877 888 10.30773/pi.2019.0106 PMC6933139 31805761

[59] Patten S B Marrie R A Carta M G Depression in multiple sclerosis Int Rev Psychiatry 2017 29 05 463 472 10.1080/09540261.2017.1322555 28681616

[60] Marrie R A Reingold S Cohen J The incidence and prevalence of psychiatric disorders in multiple sclerosis: a systematic review Mult Scler 2015 21 03 305 317 10.1177/1352458514564487 25583845 PMC4429164

[61] Chwastiak L A Ehde D M Psychiatric issues in multiple sclerosis Psychiatr Clin North Am 2007 30 04 803 817 10.1016/j.psc.2007.07.003 17938046 PMC2706287

[62] Korostil M Feinstein A Anxiety disorders and their clinical correlates in multiple sclerosis patients Mult Scler 2007 13 01 67 72 10.1177/1352458506071161 17294613

[63] Goretti B Viterbo R G Portaccio E Anxiety state affects information processing speed in patients with multiple sclerosis Neurol Sci 2014 35 04 559 563 10.1007/s10072-013-1544-0 24072658

[64] Morrow S A Rosehart H Pantazopoulos K Anxiety and Depressive Symptoms Are Associated With Worse Performance on Objective Cognitive Tests in MS J Neuropsychiatry Clin Neurosci 2016 28 02 118 123 10.1176/appi.neuropsych.15070167 26569152

[65] Ribbons K Lea R Schofield P W Lechner-Scott J Anxiety Levels Are Independently Associated With Cognitive Performance in an Australian Multiple Sclerosis Patient Cohort J Neuropsychiatry Clin Neurosci 2017 29 02 128 134 10.1176/appi.neuropsych.16050085 27899051

[66] Comorbidity and Cognition in Multiple Sclerosis (CCOMS) Study Group Marrie R A Patel R Figley C R Diabetes and anxiety adversely affect cognition in multiple sclerosis Mult Scler Relat Disord 2019 27 27 164 170 10.1016/j.msard.2018.10.018 30384203

[67] Veauthier C Sleep disorders in multiple sclerosis. Review Curr Neurol Neurosci Rep 2015 15 05 21 10.1007/s11910-015-0546-0 25773000

[68] Hughes A J Turner A P Alschuler K N Association Between Sleep Problems and Perceived Cognitive Dysfunction Over 12 Months in Individuals with Multiple Sclerosis Behav Sleep Med 2018 16 01 79 91 10.1080/15402002.2016.1173553 27167969

[69] Ayache S S Chalah M A Fatigue in multiple sclerosis - Insights into evaluation and management Neurophysiol Clin 2017 47 02 139 171 10.1016/j.neucli.2017.02.004 28416274

[70] Nunnari D De Cola M C D'Aleo G Rifici C Russo M Sessa E 10.1155/2015/519785 PMC4377448 25861633

[71] Morrow S A Weinstock-Guttman B Munschauer F E Hojnacki D Benedict R H Subjective fatigue is not associated with cognitive impairment in multiple sclerosis: cross-sectional and longitudinal analysis Mult Scler 2009 15 08 998 1005 10.1177/1352458509106213 19667024

[72] Hanken K Eling P Hildebrandt H Is there a cognitive signature for MS-related fatigue? Mult Scler 2015 21 04 376 381 10.1177/1352458514549567 25257615

[73] Andreasen A K Iversen P Marstrand L Siersma V Siebner H R Sellebjerg F Structural and cognitive correlates of fatigue in progressive multiple sclerosis Neurol Res 2019 41 02 168 176 10.1080/01616412.2018.1547813 30513278

[74] Pan H Wang Y Wang X Yan C Dimethyl fumarate improves cognitive impairment by enhancing hippocampal brain-derived neurotrophic factor levels in hypothyroid rats BMC Endocr Disord 2022 22 01 188 10.1186/s12902-022-01086-4 35869475 PMC9306081

[75] Fahmy E M Elfayoumy N M Abdelalim A M Sharaf S A Ismail R S Elshebawy H Relation of serum levels of homocysteine, vitamin B12 and folate to cognitive functions in multiple sclerosis patients Int J Neurosci 2018 128 09 835 841 10.1080/00207454.2018.1435538 29384421

[76] Giazkoulidou A Messinis L Nasios G Cognitive functions and social cognition in multiple sclerosis: An overview Hell J Nucl Med 2019 22 22, Suppl)102 110 30877728

[77] Lin X Zhang X Liu Q Social cognition in multiple sclerosis and its subtypes: A meta-analysis Mult Scler Relat Disord 2021 52 52 102973 10.1016/j.msard.2021.102973 33962135

[78] Doskas T Vavougios G D Karampetsou P Neurocognitive impairment and social cognition in multiple sclerosis Int J Neurosci 2021;(25 1 16 10.1080/00207454.2021.1879066 33527857

[79] van der Hiele K van Egmond E EA Jongen P J Empathy in multiple sclerosis--Correlates with cognitive, psychological and occupational functioning Mult Scler Relat Disord 2020 41 41 102036 10.1016/j.msard.2020.102036 32169828

[80] Dulau C Deloire M Diaz H Social cognition according to cognitive impairment in different clinical phenotypes of multiple sclerosis J Neurol 2017 264 04 740 748 10.1007/s00415-017-8417-z 28220288

[81] Neuhaus M Bagutti S Yaldizli Ö Characterization of social cognition impairment in multiple sclerosis Eur J Neurol 2018 25 01 90 96 10.1111/ene.13457 28898535

[82] Bisecco A Altieri M Santangelo G Resting-State Functional Correlates of Social Cognition in Multiple Sclerosis: An Explorative Study Front Behav Neurosci 2020 13 03 276 10.3389/fnbeh.2019.00276 32116584 PMC7016209

[83] Argento O Spanò B Serra L Relapsing-remitting and secondary-progressive multiple sclerosis patients differ in decoding others' emotions by their eyes Eur J Neurol 2022 29 02 505 514 10.1111/ene.15155 34687120

[84] Parada-Fernández P Oliva-Macías M Amayra I Accuracy and reaction time in recognition of facial emotions in people with multiple sclerosis Rev Neurol 2015 61 10 433 440 26553173

[85] Luminet O Bagby R M Taylor G J. Alexithymia: Advances in Theory, Research & Clinical Practice. Cambridge: Cambridge University Press;2018

[86] Raimo S Trojano L Pappacena S Neuropsychological correlates of theory of mind deficits in patients with multiple sclerosis Neuropsychology 2017 31 07 811 821 10.1037/neu0000372 28406663

[87] Chalah M A Ayache S S Alexithymia in multiple sclerosis: A systematic review of literature Neuropsychologia 2017 104 104 31 47 10.1016/j.neuropsychologia.2017.07.034 28764994

[88] Moll J Zahn R de Oliveira-Souza R Krueger F Grafman J Opinion: the neural basis of human moral cognition Nat Rev Neurosci 2005 6 10 799 809 10.1038/nrn1768 16276356

[89] Gleichgerrcht E Tomashitis B Sinay V The relationship between alexithymia, empathy and moral judgment in patients with multiple sclerosis Eur J Neurol 2015 22 09 1295 1303 10.1111/ene.12745 26095629

[90] Patil I Young L Sinay V Gleichgerrcht E Elevated moral condemnation of third-party violations in multiple sclerosis patients Soc Neurosci 2017 12 03 308 329 10.1080/17470919.2016.1175380 27053464

[91] Lotto L Manfrinati A Sarlo M A new set of moral dilemmas: Norms for moral acceptability, decision times, and emotional salience J. Behav. Dec. Making 2014 27 01 57 65

[92] Realmuto S Dodich A Meli R Moral Cognition and Multiple Sclerosis: A Neuropsychological Study Arch Clin Neuropsychol 2019 34 03 319 326 10.1093/arclin/acy047 29850776

[93] McCabe M P Stokes M McDonald E Changes in quality of life and coping among people with multiple sclerosis over a 2 year period Psychol Health Med 2009 14 01 86 96 10.1080/13548500802017682 19085315

[94] Gutbrod K Krouzel C Hofer H Müri R Perrig W Ptak R Decision-making in amnesia: do advantageous decisions require conscious knowledge of previous behavioural choices? Neuropsychologia 2006 44 08 1315 1324 10.1016/j.neuropsychologia.2006.01.014 16513144

[95] Neuhaus M Calabrese P Annoni J M. Decision-Making in Multiple Sclerosis Patients: A Systematic Review. Mult Scler Int. 2018; ( 2018):7835952. DOI:10.1155/2018/7835952 PMC5867562 29721338

[96] Damasio A R Descartes Error: Emotion, Reason, and the Human Brain New York:Grosset Putmann;1994

[97] Kleeberg J Bruggimann L Annoni J M van Melle G Bogousslavsky J Schluep M Altered decision-making in multiple sclerosis: a sign of impaired emotional reactivity? Ann Neurol 2004 56 06 787 795 10.1002/ana.20277 15468096

[98] Simioni S Schluep M Bault N Multiple sclerosis decreases explicit counterfactual processing and risk taking in decision making PLoS One 2012 7 12 e50718 10.1371/journal.pone.0050718 23227201 PMC3515609

[99] Hoffmann J A von Helversen B Rieskamp J Pillars of judgment: how memory abilities affect performance in rule-based and exemplar-based judgments J Exp Psychol Gen 2014 143 06 2242 2261 10.1037/a0037989 25285427

[100] Parker A M de Bruin W B Fischhoff B Weller J Robustness of Decision-Making Competence: Evidence from two measures and an 11-year longitudinal study J Behav Decis Making 2018 31 03 380 391 10.1002/bdm.2059 PMC6075651 30083026

[101] Hoffmann J A Bareuther L Schmidt R Dettmers C The relation between memory and decision-making in multiple sclerosis patients Mult Scler Relat Disord 2020 37 37 101433 10.1016/j.msard.2019.101433 32173000

[102] Zamarian L Berger T Pertl M T Decision making and framing effects in multiple sclerosis Eur J Neurol 2021 28 04 1292 1298 10.1111/ene.14669 33296528 PMC7986618

[103] Pettigrew C Soldan A Defining Cognitive Reserve and Implications for Cognitive Aging Curr Neurol Neurosci Rep 2019 19 01 1 10.1007/s11910-019-0917-z 30627880 PMC7812665

[104] Sumowski J F Leavitt V M Cognitive reserve in multiple sclerosis Mult Scler 2013 19 09 1122 1127 10.1177/1352458513498834 23897894

[105] Argento O Nocentini U. Neuropsychological assessment in multiple sclerosis. In Ayman El-Baz and Jasjit S Suri. Neurological Disorders and Imaging Physics, Volume 2; Engineering and clinical perspectives of multiple sclerosis. Bristol: IOP ebooks; 2019. 242-264

[106] Langdon D W Amato M P Boringa J Recommendations for a Brief International Cognitive Assessment for Multiple Sclerosis (BICAMS) Mult Scler 2012 18 06 891 898 10.1177/1352458511431076 22190573 PMC3546642

[107] Erlanger D M Kaushik T Caruso L S Reliability of a cognitive endpoint for use in a multiple sclerosis pharmaceutical trial J Neurol Sci 2014 340(1-2):123 129 10.1016/j.jns.2014.03.009 24656433

[108] Pardini M Uccelli A Grafman J Yaldizli Ö Mancardi G Roccatagliata L Isolated cognitive relapses in multiple sclerosis J Neurol Neurosurg Psychiatry 2014 85 09 1035 1037 10.1136/jnnp-2013-307275 24686566

[109] van Oirschot P Heerings M Wendrich K den Teuling B Martens M B Jongen P J Symbol Digit Modalities Test Variant in a Smartphone App for Persons With Multiple Sclerosis: Validation Study JMIR Mhealth Uhealth 2020 8 10 e18160 10.2196/18160 33016886 PMC7573704

[110] National MS Society Cognition Work Team Wojcik C M Beier M Costello K Computerized neuropsychological assessment devices in multiple sclerosis: A systematic review Mult Scler 2019 25 14 1848 1869 10.1177/1352458519879094 31637963 PMC6875828

[111] Benedict R H Munschauer F Linn R Screening for multiple sclerosis cognitive impairment using a self-administered 15-item questionnaire Mult Scler 2003 9 01 95 101 10.1191/1352458503ms861oa 12617275

[112] Chen M H Goverover Y Genova H M DeLuca J Cognitive Efficacy of Pharmacologic Treatments in Multiple Sclerosis: A Systematic Review CNS Drugs 2020 34 06 599 628 10.1007/s40263-020-00734-4 32361940 PMC7275014

[113] Landmeyer N C Bürkner P C Wiendl H Disease-modifying treatments and cognition in relapsing-remitting multiple sclerosis: A meta-analysis Neurology 2020 94 22 e2373 e2383 10.1212/WNL.0000000000009522 32430312

[114] DeLuca J Chiaravalloti N D Sandroff B M Treatment and management of cognitive dysfunction in patients with multiple sclerosis Nat Rev Neurol 2020 16 06 319 332 10.1038/s41582-020-0355-1 32372033

[115] Chen M H Chiaravalloti N D DeLuca J Neurological update: cognitive rehabilitation in multiple sclerosis J Neurol 2021 268 12 4908 4914 10.1007/s00415-021-10618-2 34028615

[116] Tacchino A Pedullà L Bonzano L A New App for At-Home Cognitive Training: Description and Pilot Testing on Patients with Multiple Sclerosis JMIR Mhealth Uhealth 2015 3 03 e85 10.2196/mhealth.4269 26323749 PMC4704979

[117] Gómez-Gastiasoro A Peña J Ibarretxe-Bilbao N Lucas-Jiménez O Díez-Cirarda M Rilo O 10.1155/2019/4647134 PMC6854258 31772682

[118] Gharakhanlou R Wesselmann L Rademacher A Exercise training and cognitive performance in persons with multiple sclerosis: A systematic review and multilevel meta-analysis of clinical trials Mult Scler 2021 27 13 1977 1993 10.1177/1352458520917935 32390502

